# A comparison of the brainstem auditory evoked response in healthy ears of unilaterally deaf dogs and bilaterally hearing dogs

**DOI:** 10.1007/s11259-016-9668-3

**Published:** 2016-11-29

**Authors:** M. Plonek, J. Nicpoń, K. Kubiak, M. Wrzosek

**Affiliations:** 1 Department of Internal Diseases with Clinic for Horses, Dogs and Cats, The Faculty of Veterinary Medicine, Wroclaw University of Environmental and Life Sciences, pl. Grunwaldzki 47, 50-366 Wrocław, Poland; 2 Centre for Experimental Diagnostics and Biomedical Innovations, Grunwaldzki sq. 47, 50-366 Wroclaw, Poland

**Keywords:** Subcortical auditory plasticity, Congenital sensorineural deafness, Amplitude, Latency

## Abstract

**Aims:**

Auditory plasticity in response to unilateral deafness has been reported in various animal species. Subcortical changes occurring in unilaterally deaf young dogs using the brainstem auditory evoked response have not been evaluated yet. The aim of this study was to assess the brainstem auditory evoked response findings in dogs with unilateral hearing loss, and compare them with recordings obtained from healthy dogs.

**Methods:**

Brainstem auditory evoked responses (amplitudes and latencies of waves I, II, III, V, the V/I wave amplitude ratio, wave I-V, I-III and III-V interpeak intervals) were studied retrospectively in forty-six privately owned dogs, which were either unilaterally deaf or had bilateral hearing. The data obtained from the hearing ears in unilaterally deaf dogs were compared to values obtained from their healthy littermates.

**Results:**

Statistically significant differences in the amplitude of wave III and the V/I wave amplitude ratio at 75 dB nHL were found between the group of unilaterally deaf puppies and the control group. The recordings of dogs with single-sided deafness were compared, and the results showed no statistically significant differences in the latencies and amplitudes of the waves between left- (AL) and right-sided (AR) deafness.

**Conclusions:**

The recordings of the brainstem auditory evoked response in canines with unilateral inborn deafness in this study varied compared to recordings from healthy dogs. Future studies looking into electrophysiological assessment of hearing in conjunction with imaging modalities to determine subcortical auditory plasticity and auditory lateralization in unilaterally deaf dogs are warranted.

## Introduction

The peripheral auditory system serves to change sound wave energy into electrical impulses, and animals have learnt to use it primarily for communication in the course of evolution. Canine congenital sensorineural deafness occurs in at least 90 breeds of dogs, predominantly due to cochleosaccular degeneration taking place within six weeks after birth (Strain 1996). The mode of inheritance of deafness in predisposed breeds has not yet been established although autosomal recessive, autosomal pleiotropic recessive and polygenic mechanisms have been suggested. Many of these studies closely associate hearing impairment with pigmentation (Strain 2004). Recent efforts to sequence the canine genome seem promising in identifying the gene(s) responsible for this defect in affected dogs (Strain 2015). Similar studies concerning canine early-onset presbycusis are being carried out (Yokoyama et al. 2012). Several studies have suggested a higher incidence of unilateral deafness than bilateral hearing loss in affected breeds (Holliday et al. 1992; Rak and Distl 2005; Strain 2004).

The recording of the brainstem auditory evoked response (BAER) has proven useful in evaluating auditory function both in humans and animals due to its objectivity, cost and time effectiveness as well as non-invasiveness (Wilson and Mills 2005). It is a summation of electrical activity generated by various structures within the auditory system in response to repeated acoustic stimuli, and it is used in animals to assess hearing and brainstem function. It enables identifying the site of lesion within the auditory pathway and estimating the hearing threshold (Bodenhamer et al. 1985; Holliday and Te Selle 1985). The responses are measured using four subcutaneous electrodes positioned at the midline of the scalp, at the mastoid processes near the base of the ears and on the neck. Stimuli are delivered via headphones or insert earphones. Various BAER protocols have been described in canines and vary depending on the type of the stimulus type (click vs tone burst), its intensity (measured in decibels – sound pressure level, hearing level and sensation level (Roeser RJ 2007)), rate, polarity (rarefaction, condensation or alternating) and the type of stimulator used (Wilson and Mills 2005).

Studies conducted on unilaterally deaf cats and guinea pigs have revealed profound functional changes in the structure and function of the central auditory system which modify the asymmetric cortical response patterns of the auditory cortex (Syka 2002). Subcortical plasticity, on the other hand, has only been studied in human subjects with unilateral earplug induced auditory deprivation (Decker and Howe 1981; Maslin et al. 2013; Munro and Blount 2009).

Unilateral deafness has been reported to occur more commonly than bilateral deafness in numerous groups of studied breeds of dogs, including Dalmatians (Holliday et al. 1992), border collies (Platt et al. 2006), English setters, Australian cattle dogs and bull terriers (Strain 2004). To the authors’ best knowledge, a comparison of the functional BAER assessment of unilaterally deaf dogs and those with normal hearing has not been performed to date.

The aim of this study was to compare the BAER recorded at 75, 90 and 105 dB nHL with reference to the parameters of waves I, II, III and V in the healthy ears of unilaterally deaf dogs with values obtained from dogs with normal bilateral hearing. We hypothesized that there would be differences in the BAER recordings of unilaterally deaf dogs, which could suggest their hearing was better compared with healthy dogs.

## Methods

### Animals

The study was conducted retrospectively on a group of unilaterally deaf dogs (group A) and a control group consisting of neurologically intact animals (group B) which were patients at the Department of Internal Medicine and Clinic of the Diseases of Horses, Dogs and Cats at the Wroclaw University of Environmental and Life Sciences. Hence, Local Ethics approval was not required. All dogs were purebreds predisposed to deafness (bull terriers, English setters, Australian cattle dogs and the Dogo Argentino) and underwent the BAER test as part of a screening program to exclude deaf individuals from breeding. Table 1 summarizes the animals included in the two study groups. Group A was further divided into two subgroups, comprising fourteen right-sided (group AR) and nine left-sided (group AL) unilaterally deaf dogs. All the owners were questioned about the dog’s history. Any dogs with previous otitis and/or any administration of ototoxic drugs were excluded from the study. All the patients underwent physical and neurological examinations.

**Table 1:** Summary of the experimental groups and their data

Experimental groups	A (study)	B (control)
Number of animals	23	23
Median age	8 weeks	8 weeks
Sex	11 females, 12 males	14 females, 9 males
Breeds of dogs	bullterrier (9) , English setter (5), Australian cattle dog (2), Dogo Argentino (7)	bullterrier (8), English setter (4), Australian cattle dog (4), Dogo Argentino (7)
Unilateral left-sided deafness	9	
Unilateral right-sided deafness	14	

### Neurodiagnostic testing

The recording was carried out in a quiet room, under sedation, using medetomidine (10 μg/kg i.m., Narcostart 1 mg/ml, 10 ml, Animedica Polska sp. z o.o., Chwaszczyńska 198a 81–571 Gdynia, Poland) and butorphanol (0.1 mg/kg i.m, Torbugesic 10 mg/ml 10 ml, Pfizer Trading Polska Sp zo.o., Postępu 17B, 02–676 Warsaw, Poland). Prior to the recording, an otoscopic examination was conducted to minimise the possibility of conductive problems. The tympanic membrane was visualized and was intact in all the cases. Any excess debris was removed manually using cotton tipped applicators prior to the BAER recording.

Patients were positioned in sternal recumbency, and their oxygen levels were monitored through a pulse oxymeter. Four stainless steel needle electrodes (12 mm subdermal needles,) were placed subcutaneously. The negative electrodes were placed near the mastoid prominence of each ear. A recording electrode was inserted at the midline of the head at the vertex (positive), and a ground electrode was placed at the midline of the neck.

After completing the recordings, sedation was reversed using atipamezole at a dose of 10 μg/kg i.m or to effect (Antisedan 5 mg/1 ml, Orion Corporation Orionintie 1 FIN- 02200 Espoo Finland).

### Equipment

A Nicolet Viasys Viking Quest evoked potential system was used. Stimuli were provided through auditory tubal insert earphones (10 mm, Carefusion, Germany). Rarefaction stimulation was applied at 11 Hz, and recordings were made at 75, 90 and 105 dB nHL (dB). A masking noise with an offset of −20 dB from the test intensity was used to eliminate the crossover effect (Goncalves et al. 2008) (high stimulus intensities directed at the test ear can stimulate the cochlea of the non-test ear Wilson and Mills 2005). The broadband click included frequencies between 150 Hz and 3 kHz. In all the dogs, the left ear was tested first. The results of 300 recordings were averaged. Wave amplitudes and latencies were marked manually.

### Data classification

Four waves (I, II, III, V) were identified and analyzed in the study. The tracings were analyzed for wave I, II, III and V amplitude and latency, wave V to I amplitude ratio and interpeak intervals between waves I-V, I-III and III-V. Patients were deemed unilaterally deaf if no waveform was identifiable on the tracings at any of the three stimulus intensities, and the side of deafness was noted.

The amplitude of each wave was determined as the difference in the value of the maximum of each peak and the minimum of the trough following it. The latency was established as the peak latency. Latencies (measured in milliseconds) and amplitudes (measured in microvolts) of waves I, II, III and V were measured to the nearest hundredth and recorded manually. All measurements were carried out independently by two individuals (MP, MW). Two dogs did not have a BAER recording at 105 dB due to a technical error.

### Comparison of recordings depending on the side of deafness

Group A was further divided into two subgroups depending on the side of the unilateral deafness (AR – unilateral right-sided deafness; AL – unilateral left sided deafness). The values of the above described waves were compared between the two subgroups.

### Statistical methods

The normality of data distribution was analysed using the Shapiro-Wilk test. The Student’s t test was used to compare parametric data, whereas the non-parametric Mann-Whitney U test was used to compare wave amplitude and latency variables that were not distributed normally (StatSoft. Inc. 2011 STATISTICA (data analysis software system, version 10)). The amplitude of wave V at 75 dB and of waves II and III at 105 dB were parametric. The latencies of wave III and V, the wave I-V, I-III and III-V interval at 75 dB and of wave V, the I-V and III-V interval at 90 dB were also parametric. The remaining data were non-parametric. Statistical significance was determined at *p* < 0.05.

## Results

### Wave amplitudes

**Fig. 1:**
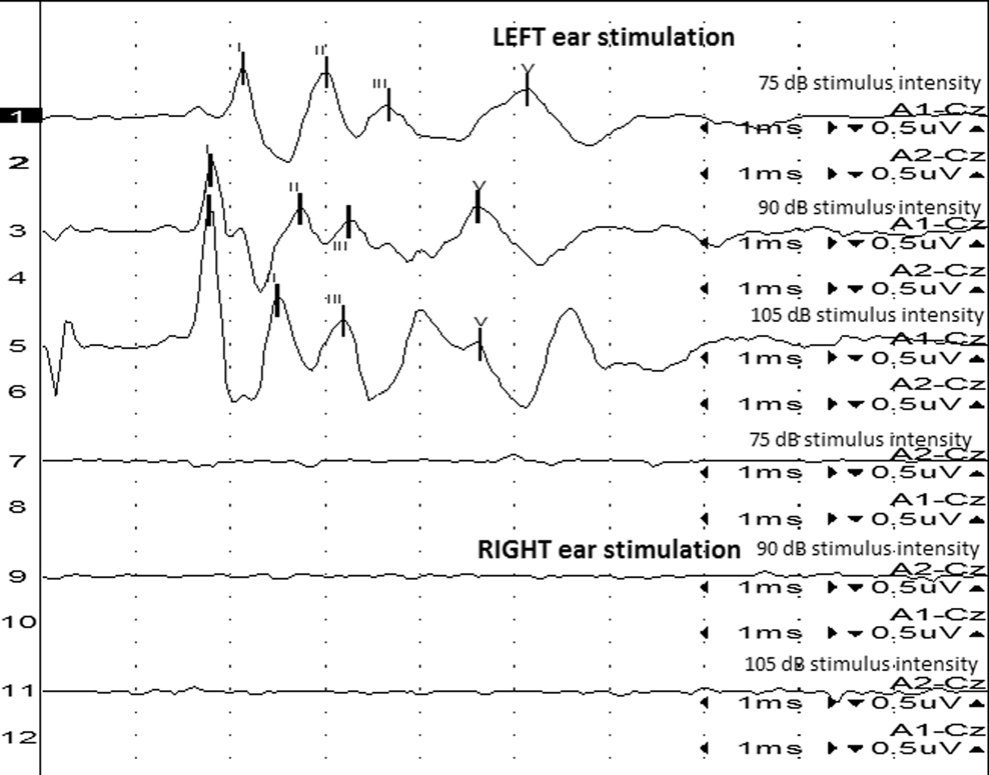
A BAER obtained from a unilaterally deaf English setter puppy

**Fig. 2:**
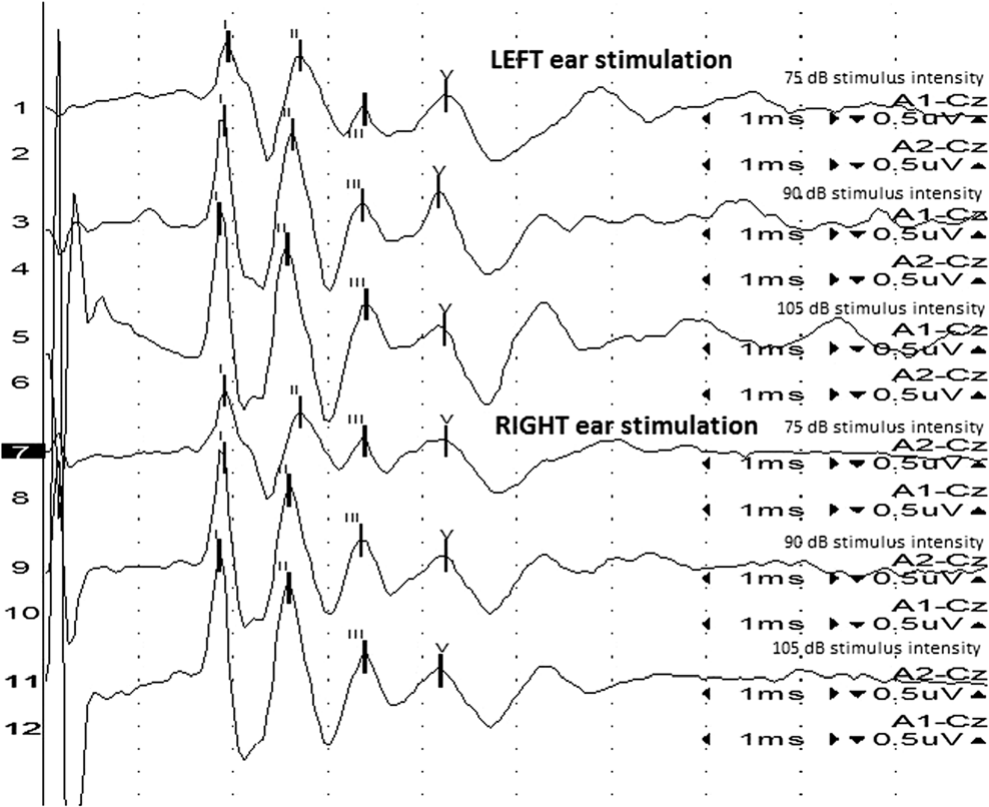
A BAER from a bull terrier puppy with bilaterally normal hearing

**Fig. 3:**
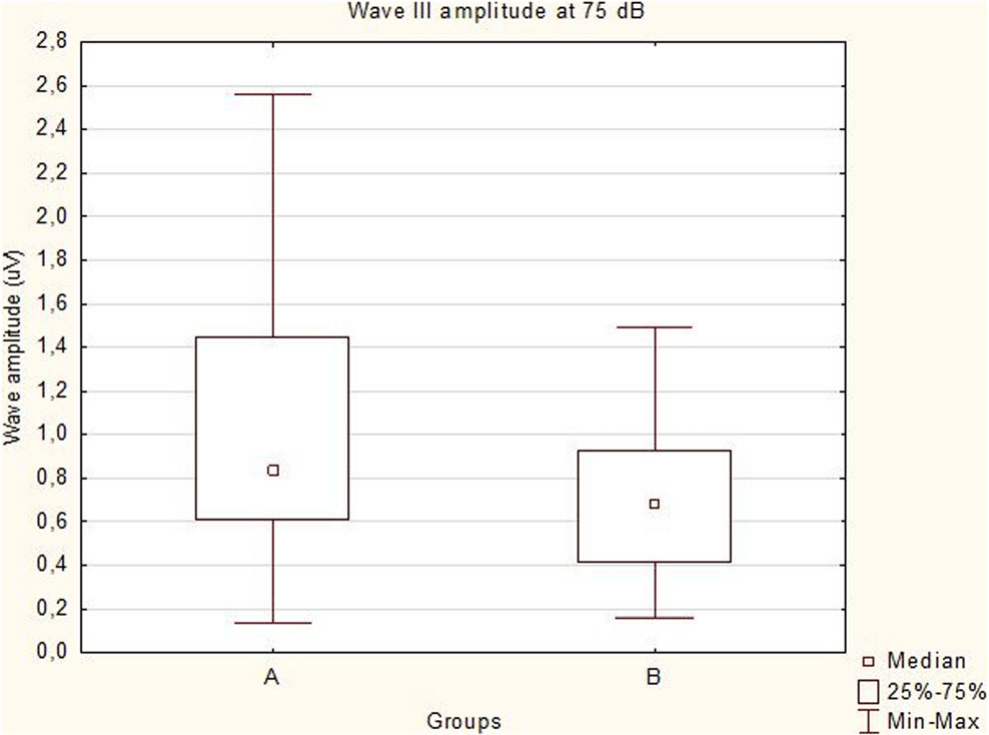
Box and whisker plot showing data distribution of wave III amplitudes at 75 dB

**Fig. 4:**
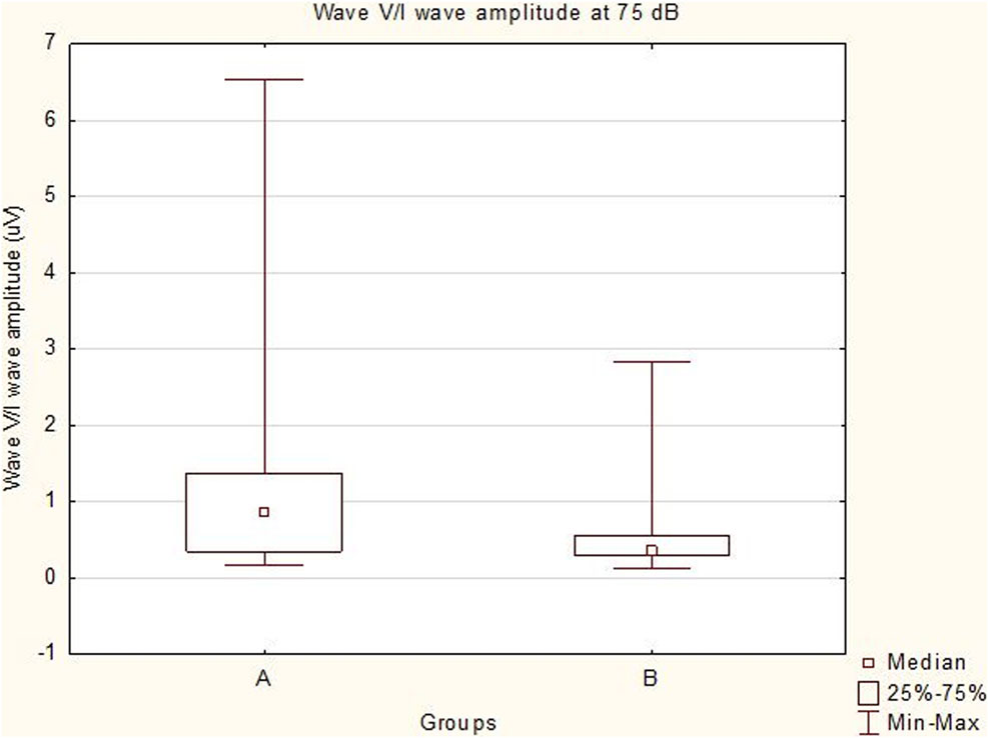
Box and whisker plot showing data distribution of wave V/I amplitude ratio at 75 dB

All the recorded waves were well defined and identifiable by the authors (MP, MW, Figs. 1 and 2). A statistical significance was noted between the amplitudes of wave III in groups A and B (*p* < 0.05) at 75 dB, with the mean amplitudes higher in group A (1.03 vs 0.70 μV, Fig. 3). A statistical significance was also noted between the V/I wave amplitude ratio at that sound intensity (Fig. 4). A higher wave V/I amplitude ratio was present in group A (1.22 vs 0.61). No other statistical differences were noted for wave amplitudes. All recorded wave amplitudes and latencies are presented as mean and standard deviations in Tables 2 and 3.

**Table 2:** Mean and standard deviation (SD) values of wave amplitudes (uV) in dogs with unilateral deafness (group A) and control dogs (group B)

		75 dB AMP I	75 dB AMP II	75 dB AMP III	75 dB AMP V	V/I Amplitude Ratio	90 dB AMP I	90 dB AMP II	90 dB AMP III	90 dB AMP V	V/I Amplitude Ratio	105 dB AMP I	105 dB AMP II	105 dB AMP III	105 dB AMP V	V/I Amplitude Ratio
Group A (*n* = 23)	Mean	1.92	1.83	1.03	1.14	1.22	3.70	2.38	1.81	1.49	0.54	5.35	4.04	2.49	2.38	0.46
	SD	1.65	1.30	0.61	0.45	1.42	1.99	2.07	1.58	1.00	0.56	1.41	1.94	1.43	2.10	0.39
Group B (*n* = 23)	Mean	2.31	1.62	0.70	0.91	0.61	3.94	2.43	1.05	1.14	0.30	5.49	3.70	2.07	1.78	0.35
	SD	1.42	0.86	0.38	0.45	0.63	1.58	1.69	0.67	0.69	0.13	1.77	2.32	1.16	0.71	0.15

**Table 3:** Mean and standard deviation (SD) values of wave latencies (ms) in dogs with unilateral deafness (group A) and control dogs (group B)

		75 dB LAT I	75 dB LAT II	75 dB LAT III	75 dB LAT V	Wave 1-V interval	Wave I-III interval	Wave III-V interval
Group A (*n* = 23)	Mean	1.94	2.72	3.39	4.51	2.57	1.45	1.12
	SD	0.17	0.32	0.34	0.44	0.39	0.26	0.45
Group B (*n* = 23)	Mean	1.95	2.8	3.47	4.54	2.59	1.52	1.07
	SD	0.13	0.26	0.33	0.44	0.35	0.27	0.45
		90 dB LAT I	90 dB LAT II	90 dB LAT III	90 dB LAT V	Wave 1-V interval	Wave I-III interval	wave III-V interval
Group A (*n* = 23)	Mean	1.85	2.67	3.37	4.39	2.54	1.52	1.02
	SD	0.14	0.2	0.33	0.37	0.36	0.29	0.3
Group B (*n* = 23)	Mean	1.89	2.7	3.49	4.43	2.53	1.59	0.94
	SD	0.12	0.22	0.27	0.36	0.28	0.23	0.39
		105 dB LAT I	105 dB LAT II	105 dB LAT III	105 dB LAT V	Wave 1-V interval	Wave I-III interval	wave III-V interval
Group A (*n* = 23)	Mean	1.8	2.54	3.31	4.21	2.4	1.51	0.9
	SD	0.03	0.18	0.3	0.28	0.28	0.29	0.25
Group B (*n* = 23)	Mean	1.8	2.59	3.44	4.28	2.49	1.64	0.85
	SD	0.03	0.11	0.23	0.26	0.26	0.22	0.3

### Wave latencies

No statistically significant differences in wave latencies were recorded at 75, 90 and 105 dB.

### Comparison of unilaterally left and right-sided deaf dogs

There were no statistically significant differences in the wave amplitudes and latencies at 75, 90 and 105 dB between dogs with left and right-sided unilateral deafness.

## Discussion

The occurrence of a canine congenital sensorineural deafness has been described in literature and was suspected in the present study population. Histologically, this type of deafness is characterized by a cochleosaccular end organ degeneration. The stria vascularis degeneration, a collapse of Reissner’s membrane and the cochlear duct, hair cell degeneration in the organ of Corti and a collapse of the saccule are followed by a loss of spiral ganglion cells (Strain 2015). These changes are permanent (Strain 1996). The mean age of the studied dogs was eight weeks. At this age, the auditory response is fully matured (Poncelet et al. 2000; Strain et al. 1991) and it is possible to diagnose sensorineural deafness.

In humans, the neural generators of BAER waves are believed to originate in more than one anatomic structure (Hall 1992; Møller 1998). It has been acknowledged that wave I originates in the auditory nerve while wave II reflects the activity of the proximal VIII cranial nerve or cochlear nucleus (Strain 2011b). Wave III is thought to originate in the lower pons – superior olivary complex and waves IV and V in the mid or upper pons or inferior colliculus (Chiappa and Hill 1997). There has been inconsistency regarding wave labeling in dogs. Different electrode configurations can elicit varying numbers of positive peaks (Kawasaki and Inada 1994), which have been labeled differently in literature (wave I (Holliday and Te Selle 1985; Kawasaki and Inada 1994), Ia (Bodenhamer et al. 1985), Ib (Bodenhamer et al. 1985), II (Kawasaki and Inada 1994), IIa (Holliday and Te Selle 1985), IIb (Holliday and Te Selle 1985), III (Kawasaki and Inada 1994), IIIa (Holliday and Te Selle 1985), IIIb (Holliday and Te Selle 1985), the wave III-IV complex (Venker-van Haagen et al. 1989), IV (Kawasaki and Inada 1994), IV (Strain et al. 1991), V (Kawasaki and Inada 1994), Vb (Myers et al. 1985).

The BAERs present waves generated from CN VIII and the auditory pathway to the lateral lemniscus (Legatt 2012) or caudal colliculus (Shiu et al. 1997) (there is still no general agreement in both human and animal medicine, whether the BAER includes the caudal colliculus). The exact generators of waves III-V are difficult to determine as they arise from multiple structures of the brainstem. It is thought that the main generator of wave III is the trapezoid body (Scherg and von Cramon 1985) or the superior olivary complex (Strain 2011d). In humans, wave IV seems to reflect activity in the dorsal and rostral pons, whereas animal studies have shown it to be predominantly generated in the nucleus of the lateral lemniscus (Legatt 2012). Studies on humans are inconclusive regarding the exact wave V generator. It seems to arise at the inferior colliculus itself or at the rostral portion of the lateral lemniscus before terminating in the inferior colliculus (Kimiskidis et al. 2004).

In 1991, Moore found that human neonatal unilateral hearing loss leads to a rearrangement of binaural connections in the auditory midbrain and collicular neurons in response to stimulation of the intact ear (Moore 1991). Such changes were not observed in individuals suffering from adult hearing loss. Therefore, carrying out the present study on a group of young canines in order to determine BAER differences seems justifiable. Other studies conducted on unilaterally deafened cats and gerbils showed altered responses and thresholds of activation in auditory pathways ipsilateral to the intact ear, with little change occurring in the contralateral pathways in animals six months to one year after cochlear ablation (Kitzes 1984; Reale et al. 1987). Hence, it would be interesting to study the same individuals up to a year after the first BAER assessment in order to compare the recordings.

This study showed a statistical significance between wave III amplitudes in the two study groups at 75 dB, with a higher amplitude of this wave recorded in unilaterally deaf dogs. This study protocol was standardized for all dogs, and electrode placement should not have varied between individuals. Other factors influencing the BAER wave amplitudes, such as the click repetition rate, body temperature and electrode placement are unlikely to have influenced the results because the study was carried out uniformly in all the dogs. However, it is impossible to exclude a small electrode displacement or a varying depth of subcutaneous electrode insertion between individuals. There is disagreement among authors regarding the effect of head size on the brainstem auditory evoked response. Meij et al. (1992) found that body weight and cranial distance led to an increase in wave latency in dogs, while Kemper et al. (2013) found that canine head size did not affect clinical BAER results in terms of the waveform morphology, wave latency or hearing sensitivity. In humans, head size (in addition to gender) has been found to affect the latency of waves I, III, V and the I-III and the I-V inter-peak interval (Aoyagi et al. 1990; Dempsey et al. 1986; Trune et al. 1988) as well as amplitude changes of waves I, III and V (Aoyagi et al. 1990). Few studies have focused on the variability of wave amplitudes in dogs of different sizes although it is known that small dogs have larger amplitudes than large dogs. Strain suggests amplitudes should be compared between individuals of the same breed or the same skull size (Strain 2011a). The skull sizes of the dogs undergoing the BAER examination in this study were not recorded, but the results were compared between dogs of the same breed and the same age.

Illing et al. reported that the superior olivary complex, a wave III generator in humans, showed signs of auditory plasticity, responding to hearing impairment by expressing plasticity-related substances (Illing et al. 2000). Popelar et al. found that amplitudes of evoked potentials increased over a course of three weeks in adult guinea pigs whose hair cells were destroyed (Popelar et al. 1994). There are no available literature reports concerning the disruption of excitatory and inhibitory neurotransmitter release (which may cause wave amplitude alterations) following unilateral congenital sensorineural deafness in animals. The increased wave V/I amplitude ratio at 75 dB may indicate greater excitability of neurons A statistically significant difference in the wave III amplitude was seen only at a single sound magnitude. However, wave III amplitudes at the remaining intensities were also higher in group A than in group B. In order to confirm our findings, an analysis of BAER needs to be carried out.

As shown by Sims and Moore, increasing stimulus intensity at a constant stimulus rate decreases wave latencies in dogs, and that was observed in most dogs in group A and B in our study at all three sound intensities (Sims and Moore 1984).

Brainstem auditory wave amplitudes are not analysed in some scenarios since they may vary largely. Therefore, some authors suggested the wave V/I amplitude ratio was a more valuable parameter to assess during the BAER (Kehrle et al. 2008; Musiek 1982). In humans, Salamy et al. (1975) found the wave I amplitude to be greater in newborns than in adults, whereas Jacobson et al. (1982) reported a smaller wave V amplitude in infants compared to adults, leading to a reduced wave V/I amplitude ratio, often below 1. Despite the fact that many individuals in this study, both unilaterally deaf and from the control group, had wave V/I amplitude ratios <1.0, this may not be pathological since the wave V/I amplitude ratio has not been studied systematically in dogs or cats (Strain 2011c). Gu et al. studied the wave III/I and wave V/I amplitude ratio in patients with tinnitus and found it to be increased, suggesting greater neuronal excitability, decreased inhibition from descending projections and synaptic remodeling (Gu et al. 2012).

Our study used only the BAER to assess auditory function of unilaterally deaf and healthy dogs. The laterality of this auditory test is still currently debated. There have been contradictory reports as to the location of the BAER wave generators. Some authors claim that the BAER wave generators are located contralaterally, some report them to be ipsilateral, while others suggest both contra- and ipsilateral generator involvement (Musiek and Geurkink 1982; Oh et al. 1981; Prasher 1981). If the auditory responses are indeed lateralized, the BAER examination may not suffice to determine subcortical reorganization following unilateral deafness.

Plasticity of the auditory system is a term used to describe the reorganization of the auditory system in response to variations in the auditory input, be it physiological (postnatal development – new auditory input) or pathological (cochlear injury – decreased auditory input) (Knobel 2005). Similarly, plasticity can be divided into primary (a result of sensorineural hearing loss), secondary (reintroduction of auditory stimuli) or conditioning, where the conditioned acoustic stimulus instigates neural changes in the auditory and somatosensory cortex, the hippocampus and amygdala (Gao and Suga 2000).

The formation of new projections in the somatosensory system ipsilateral to the intact ear has been described in animals deafened unilaterally at a young age (Kitzes 1996). There have been some studies assessing map reorganization in young and adult animals. Harrison et al. suggested that auditory reorganization might occur at all the levels of the auditory system following hearing impairment. Studies are still under way to determine whether tonotopic map reorganization in response to auditory damage originates at the level of the brainstem nuclei (Harrison RV et al. 1996). How and at what level the auditory system reorganizes itself to compensate for unilateral deafness is still unclear. Synaptogenesis in the inferior colliculus has been found to be greater in neonate mammals than in adults (Harris and Woolsey 1981). Therefore, young animals may have different reorganization patterns than adult unilaterally deafened individuals.

The BAER results obtained in the present study, which compared left- and right sided unilaterally deaf dogs, showed no significant differences in the studied parameters. It would be interesting to study subcortical auditory plasticity using both BAER and fMRI depending on the side of deafness in canines with sensorineural hearing loss, especially in older dogs, in order to assess the process of subcortical reorganization occurring over a longer time period.

### Study limitations

The described results were obtained only from the mastoid reference, at three sound intensities. Hearing thresholds were not recorded for all the patients, and their tracings were not included in this study. If auditory thresholds had been recorded and compared, it might have been easier to interpret and determine differences in wave amplitudes and latencies between groups. The recordings were carried out using an average of 300 sweeps per stimulus intensity in both groups. We cannot rule out that carrying out the recordings using a greater number of sweeps may have affected the results. No structural auditory pathway assessment was carried out in this study, such as MRI tractography or a histopathological examination to confirm auditory pathway changes. Moreover, no diagnostic imaging modalities of the head were performed since all the dogs enrolled in the study were purebred and predisposed to sensorineural deafness. Finally and most importantly, the sample size was small.

## Conclusion

To the authors’ knowledge, this is the first study focusing on assessing the BAER tracings of unilaterally deaf dogs and those of bilaterally hearing animals. Numerous previous reports analysing auditory plasticity in unilaterally deaf animal models used experimentally deafened animals (Moore et al. 1997; Popelar et al. 1994; Rajan and Irvine 2010; Robertson and Irvine 1989). Our results show that there were changes in the BAER in canines with inborn deafness compared to healthy dogs. The canine model of congenital sensorineural deafness may be very useful in further neurophysiological studies using functional imaging to assess the plasticity of the auditory system.
